# *Physalis pubescens* L. branch and leaf extracts inhibit lymphoma proliferation by inducing apoptosis and cell cycle arrest

**DOI:** 10.3389/fphar.2023.1192225

**Published:** 2023-07-24

**Authors:** Li Yuan-Ce, Pang Yu-Yan, Zhang Qi, Zhang Hong-Yang, Wang Yan-Wen, Sun Yu-Mei, Zeng Guang-Zhi, Yin Jun-Lin

**Affiliations:** Key Laboratory of Chemistry in Ethnic Medicinal Resources, State Ethnic Affairs Commission and Ministry of Education, School of Ethnic Medicine, Yunnan Minzu University, Kunming, China

**Keywords:** *Physalis pubescens* L., lymphoma, physalin, cell cycle, apoptosis

## Abstract

*Physalis pubescens* L. is an annual or perennial plant in the family Solanaceae It is used in traditional medicine for treating sore throats, coughs, urinary discomfort, and astringent pain, and externally for pemphigus and eczema in northern China. The proliferation inhibitory activity and mechanisms of the ethyl acetate extract (PHY-EA) from the leaves of *Physalis pubescens* were investigated. High performance liquid chromatography was used to identify the chemical composition of PHY-EA; sulforhodamine B was used to detect the proliferation inhibitory effect of PHY-EA on MCF-7, CA-46, Hela, HepG2, B16, and other tumor cells; flow cytometry was used to detect the effect of PHY-EA on the lymphoma cell cycle and apoptosis; Western blot was used to detect the expression of the cycle- and apoptosis-related proteins. The expression of Ki-67 and cleaved caspase 3 was detected by immunohistochemistry. The results showed that PHY-EA contained physalin B, physalin O, and physalin L. PHY-EA blocked the cell cycle of G2/M→G0/G1 in lymphoma cells and induced apoptosis in tumor cells. Mouse transplantation tumor experiments showed that PHY-EA had a significant inhibitory effect on mouse transplantation tumors, and the tumor volume and weight were significantly reduced. In conclusion, PHY-EA has a good antiproliferative effect on Burkkit lymphoma, indicating its potential medicinal value.

## Introduction

Malignant lymphoma is a common malignant neoplasm originating in the lymphohematopoietic system (Munakata et al., 2019). Burkkit lymphoma is a highly aggressive form of non-Hodgkin’s B-cell lymphoma (Dolma et al., 2003). Currently, the standard first-line regimen of rituximab plus CHOP (cyclophosphamide, doxorubicin, adriamycin, vincristine, oncovin, and prednisone) is associated with an overall survival of 60%–70% at 4 years. However, 30% of patients still develop drug resistance or relapse and experience metastases, so there is an urgent need to identify new therapeutic agents to improve the survival rate of relapsed patients (Godfrey et al., 2018).

*Physalis pubescens* L. is an annual herb in the family Solanaceae, and its ripe fruits are golden yellow (Wen et al., 2020). This species is unique to northern China. *Physalis pubescens* has many ethnopharmacological applications, and its fruit, calyx, branches, and leaves possess various medicinal activities. It is widely used in the treatment of diabetes and various skin diseases In addition, *Physalis pubescens* has been found to exhibit good anti-tumor activity (Fouche et al., 2008). It has a high edible and medicinal value and contains 18 essential amino acids, 21 trace elements, and large amounts of minerals, vitamins, and unsaturated fatty acids (Wang et al., 2020). It contains steroids, flavonoids, and other chemical components, among which the compound physalin is unique in the genus *Physalis*, with anti-inflammatory (Wang et al., 2020) and anti-tumor (Fan et al., 2018) activities. Physalin exhibits good anti-tumor activity and inhibits the growth of many tumor cells, including Malhavu cells from liver cancer (Wang et al., 2016), HeLa cells from cervical cancer (Zeng et al., 2017), and prostate cancer (Ding et al., 2015). Reports on the anti-tumor mechanism of *P. pubescens* have focused on the active compound physalin B, which is the most reported physalin-like compound from *P. pubescens* and can elevate reactive oxygen species, inhibit tumor necrosis factor alpha and nuclear factor kappa beta, and activate Noxa-related apoptotic pathways, thereby inducing apoptosis (Chen et al., 2016; Xu et al., 2017; Zhao et al., 2017). It also inhibits cell proliferation by blocking the tumor cell cycle in the G2/M phase (Ma et al., 2015; Cao et al., 2019; Qiu et al., 2021; Zhang et al., 2021; Zhang et al., 2021). Physalin O and L have structures highly similar to physalin B and are abundant in *P. pubescens*. In addition, *P. pubescens* exhibits various pharmacological activities, including antibacterial (Fernandes et al., 2019), antioxidant (Fernandes et al., 2019), and antidiabetic (Chen et al., 2019) activity.

There have been many reports on the activity of physalins; however, the physalin content in *P. pubescens* has not been determined. We examined the physalin B, physalin O, and physalin L contents in the branch and leaf extracts of *P. pubescens* and found that all three compounds were present, with physalin B being the most abundant. However, studies on the pharmacological effects and active ingredients of *P. pubescens* have mainly focused on the fruit part of the calyx, and there are no studies on the pharmacological activity of the branches and leaves of *P. pubescens*. We screened the anti-tumor activity of the PHY-EA (PHY-EA) of the branches and leaves and found that PHY-EA had some anti-tumor activity against a variety of tumor cells, with the best anti-tumor activity against diffuse large B lymphoma [median inhibitory concentration (IC_50_) for the SU-DHL-4, Daudi, and CA-46 cell lines were 9.49 ± 0.12 μg/mL, 2.49 ± 0.05 μg/mL, and 4.79 ± 0.60 μg/mL, respectively).

Overall, we have identified for the first time the physalin contents in the leaves and branches of *P. pubescens*, elucidated the anti-tumor activity of *P. pubescens* branch extracts against diffuse large B lymphoma, and increased the understanding of the medicinal value of *P. pubescens*.

## Experimental materials and experimental animals

### Experimental animals

Four-to six-week-old male BALB/c nude mice were used (Hunan Slaughter Jingda Laboratory Animal Co., Ltd., China). All procedures involving animal experiments were performed in accordance with the Administration of Laboratory Animal Affairs and Ethical Guidelines for Animal Experimental Institutions of Yunnan Minzu University (2021-050).

### Experimental reagents

The cell cycle assay kits were purchased from Shanghai Yisheng Biological Co, China. Tumor cells (H2122, HUH-7, MDA-MB-231, PC3, SPC-A1, HeLa, HepG2, B16, SU-DHL-4, CA-46, and Daudi) were purchased from Kunming Cell Bank, Chinese Academy of Sciences. An apoptosis assay kit was purchased from Shanghai Yisheng Biotechnology Co, China. *Physalis pubescens* samples were collected from Yanbian City, Jilin Province, China (sample no. 20170825) and stored at the Laboratory of Targeted Drug Development and Application, Yunnan Minzu University. Physalin O, physalin L, and physalin B were purchased from Wuhan ChemFace Co, China. Primary antibodies (caspase 3, caspase 9, Cyclin-dependent kinase 2 (Cdk2), Cyclin-dependent kinase 4 (Cdk4), cleaved caspase 3, Cyclin A2,β-actin, and GADPH) and secondary antibodies against rabbits were purchased from Shanghai Protintech Co, China. SpectraMax i3x enzyme marker (Molecular Devices), A DMIL LED trinocular inverted microscope (Leica, Germany), and a flow cytometer (BECKMAN COULTER, United States) were used.

### Experimental methods

#### Preparation of PHY-EA

*Physalis pubescens* branches and leaves (10 kg) underwent extraction three times with 100% methanol and 800 g of extracts were obtained. The extracts were prepared using petroleum ether, ethyl acetate, and n-butanol. The petroleum ether (83 g), ethyl acetate (PHY-EA; 64 g), and n-butanol (103 g) extracts were then analyzed.

#### Component identification

A solution of 10 mg/mL was prepared by dissolving 100 mg of PHY-EA in 10 mL of methanol, sonicating the solution for 30 min (80 w, 100 Hz), and filtering it through a 0.45 μm microporous membrane. Physalin B, physalin O, and physalin L were prepared in 5 mg/mL solutions. The following parameters were the used: water (A)–methanol (B) gradient elution: 80%–0% A (0–80 min); isocratic elution: 100% B (80–100 min); flow rate: 1 mL/min; injection volume: 20 μL; column temperature 30°C; detection wavelength: 230 nm. A HPLC (Agilent 1260) C18 chromatographic column (ZORBAX SB-C18) was used. The compounds were analyzed using the standard curve method based on the absorbance obtained.

#### Cell recovery and passaging

The cells were quickly thawed in a water bath at 37°C. They were then resuspended by the addition of 2 mL of complete medium and centrifuged at 4°C for 4 min at 800 rpm to remove the supernatant. The cells were resuspended and cultured in 10 mL of complete medium. The suspended cells were ready for passaging when they had above 80% growth, and were centrifuged to remove the supernatant, washed three times with PBS, and resuspended in 2 mL of complete medium. Then, 200 μL of the cell suspension was added to 10 mL of complete medium and culturing was continued. For adherent cells, the old medium was aspirated and the cells were washed three times with PBS. Trypsin digestion of the cells was terminated by adding 2 mL of complete medium; then, the cells were resuspended and 200 μL of these cells were added to 10 mL of complete medium for further incubation.

#### Sulforhodamine B assay for cell proliferation

Cells were collected during the exponential growth phase, mixed into a 3 × 10^5^ cells/mL cell suspension, and 100 μL of the cell suspension was added to each well of a 96-well plate and incubated for 24 h in medium containing various concentrations of *P. pubescens* extract and 0.1% DMSO. Then, 25 μL of 80% trichloroacetic acid was added to each well and fixed for 2 h. The plates were then washed five times with distilled water. After this, 100 μL of 4% sulforhodamine B (SRB) solution was added to the wells and the cells were stained for half an hour at room temperature. The plates were then washed five times in 1% ice acetic acid, followed by the addition of 100 μL of Tris lysate per well and then the plates were shaken for 15 min.

#### Apoptosis assay

Cells were collected in 1.5 mL EP tubes, centrifuged at 4°C and 300 g for 5 min, and washed twice with PBS. Then, 100 µL of 1 × Binding Buffer was added to resuspend the cells. After this, 5 µL of Annexin V-FITC and 10 µL of propidium iodide (PI) staining solutions were added for 15 min at room temperature and protected from the light, followed by the addition of 400 µL of 1×Binding Buffer. The cells were then assayed by flow cytometry for Annexin V-FITC and PI dye absorbance.

### Cell cycle assay

Cells were centrifuged at 1,000 g for 5 min, then the supernatant was removed, samples were washed once with PBS, and the cells were collected. The cells were mixed with 1 mL of pre-cooled 70% ethanol overnight at 4°C, centrifuged at 1,000 g for 5 min, and then the precipitate was collected and washed with PBS. To this, 10 µL of PI stock solution and 10 µL of RNase A solution were added to 0.5 mL of the staining buffer, mixed well, and set aside. Propidium iodide staining solution (0.5 mL) was added to each cell sample, mixed gently, and resuspended. The cells were incubated for 30 min at 37°C in the dark, and were then detected by flow cytometry at an excitation wavelength of 488 nm.

### Western blot to detect signaling pathways

Cells were collected in 1.5 mL EP tubes, centrifuged at 1,000 rpm for 5 min at 4°C to remove the supernatant, washed twice with PBS, lysed for 10 min at 4°C with cell lysis solution, and quantified by the BCA kit. After ultrasonic breakage, the cells were heated at 98°C for 10 min to denature the proteins. These were then centrifuged at 14,000 rpm for 10 min at 4°C, and the supernatant was removed and split. Extracted protein samples were subjected to gel electrophoresis and wet transfer. Five percent skimmed milk powder was added and samples were closed for 2 h. The primary antibody was incubated overnight at 4°C (concentration of cleaved caspase 3 antibody 1:1,000; concentrations of caspase 3, caspase 9, CDK2, CDK4, CyclinA2, and GADPH, β-actin antibodies 1:3,000) and washed five times with TBST for 5 min. The secondary antibody was incubated for 2 h (secondary antibody concentration 1:7,000) and washed five times with TBST for 5 min.

### Mouse transplantation tumor experiment

Each mouse was implanted with 10^7^ Daudi cells under the skin in the axilla. Drug administration was initiated 4 days after cell implantation by peritumoral injection. Mice were divided into three groups: control, 8 mg/kg PHY-EA, and 4 mg/kg PHY-EA, which were administered for 21 days. During the treatment process, changes in body weight and tumor size were recorded, and photographic evidence was obtained at the end of the treatment.

#### Immunohistochemical assay

The tumor tissue was soaked in formalin for 24 h, rinsed for 1 h, dehydrated in ethanol, and soaked twice in xylene for 1 h. Finally, the tissue was soaked in paraffin wax three times (40 min each time) for a total of 2 h. After dehydration, the tissue was embedded in an embedding machine. The wax was placed on a microtome and cut into 4 µm thick sections and dried at 60°C for 2 h. The sections were then hydrated and the finished sections were subjected to antigen repair by placing them in an autoclave containing EDTA, steamed for 10 min, and cooled naturally. The sections were incubated with a blocking agent for 10 min at room temperature, washed three times with PBS, blocked with 10% goat serum for 1 h, washed three times with PBS, incubated with primary antibody (AKT 1:100, ERK 1:50) overnight, washed three times with PBS, incubated with secondary antibody for 20 min at room temperature, and again washed three times with PBS. Color was developed by adding DAB color developing solution for 5–10 min, then sections were stained with hematoxylin for 2 min, rinsed twice in distilled water, and dehydrated in alcohol for 10 min. The sections were sealed with gum.

### Statistical analysis

Experimental data were expressed as means ± standard errors (SE) and the data analysis was performed using SPSS 26.0 Student's t-tests were used to test the significance of differences between each dosing group and the control group, with significance indicated as * for *p* < 0.05, ** for *p* < 0.01, and *** for *p* < 0.001.

## Results

### PHY-EA contains physalin B, physalin O, and physalin L

**FIGURE 1:**
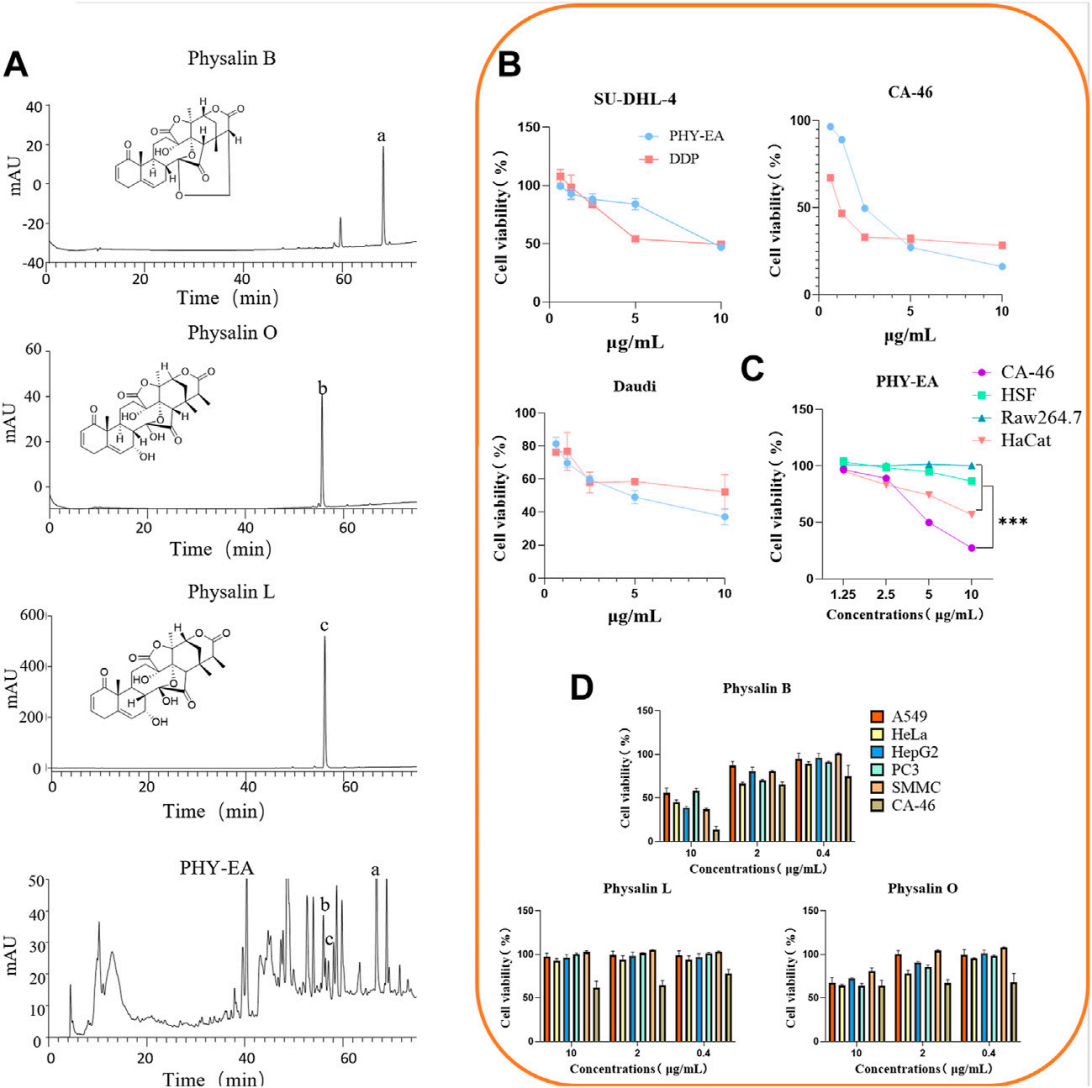
Identification of *Physalis pubescens* ethyl acetate extract (PHY-EA) components and detection of anti-tumor activity. **(A)** HPLC component analysis (*n* = 5). **(B)** Anti-proliferative activity of PHY-EA and cisplatin (DDP) against various lymphomas (*n* = 3). **(C)** Anti-proliferative activity of PHY-EA against various normal cells (*n* = 3). **(D)** Anti-proliferative activity of physalin B, O, and L against various cancer cells (*n* = 3). Data are expressed as mean ± standard error (SE), and each treatment group was compared with the control group using a Student’s *t*-test, where *p < 0.05, **p < 0.01, and***p < 0.001.

The active ingredients of PHY-EA were identified using HPLC. The retention times and peak shapes of the physalin B, physalin O, and physalin L standards were compared with those of PHY-EA to identify the components of PHY-EA. The retention times of physalin B, O, and L were 68, 55, and 56 min, respectively. The contents were 1.8% ± 0.12%, 0.3% ± 0.04%, and 0.6% ± 0.17% for physalin B, O, and L, respectively (Figure 1A; Table 1).

**TABLE 1:** High performance liquid chromatography (HPLC) determination of physalin B, L, and O contents in *Physalis pubescens* ethyl acetate extracts (PHY-EA).

Compounds	Concentrations (μg/μL)	Peak area	Calibration curves	ADM of the precision (%)	Mean recovery (%)
Physalin B (1.8% ± 0.12%)	3.00000	16,244.4	*y* = 0.0002x + 0.0132 *R* ^2^ = 0.9997	0.496	108
	1.50000	8,303.3			
	0.75000	4,020.5			
	0.37500	1,883.5			
	0.18750	927.8			
	0.09375	464.9			
Physalin L (0.6% ± 0.17%)	1.50000	16,043.20	*y* = 0.00009 x—0.0210 *R* ^2^ = 0.99948	0.213	95
	0.75000	8,397.90			
	0.37500	4,272.40			
	0.18750	2,175.00			
	0.09375	1,097.40			
Physalin O (0.3% ± 0.04%)	0.75000	3,507	*y* = 0.0002x + 0.0149 *R* ^2^ = 0.9997	0.601	95
	0.37500	1,730.6			
	0.18750	788.4			
	0.09375	361.6			
	0.04688	153			
	0.02344	73.8			

Note: the above data are from five independent experiments.

### PHY-EA has a proliferation inhibitory effect on tumor cells and normal cells

To investigate the anti-tumor activity of PHY-EA, we examined its inhibitory effects on the proliferation of 11 types of tumor cells using an SRB assay. The cell lines with an IC_50_ < 10 μg/mL were SU-DHL-4, Daudi, and CA-46 (Table 2), indicating that PHY-EA had relatively good anti-tumor activity against lymphoma cell lines. On this basis, we compared the anti-tumor activity of PHY-EA against lymphoma with that of the commonly used clinical anti-tumor drug cisplatin, and found that the IC_50_ of cisplatin against SU-DHL-4, Daudi, and CA-46 was 10.6 ± 2.864 μg/mL, 1.1 ± 0.043 μg/mL, and 8.3 ± 0.485 μg/mL, respectively, while the IC_50_ of PHY-EA against SU-DHL-4, Daudi, and CA-46 was 9.49 ± 0.12 μg/mL, 2.49 ± 0.05 μg/mL, and 4.79 ± 0.60 μg/mL, respectively. Compared with cisplatin, the IC_50_ of PHY-EA for SU-DHL-4 decreased by 12.7%, the IC_50_ for Daudi increased by 54.6%, and the IC_50_ for CA-46 decreased by 73.9%. (Figure 1B). This demonstrated that PHY-EA has a strong inhibitory effect on the proliferation of lymphoma cells *in vitro*.

**TABLE 2:** The median inhibitory concentration (IC_50_) of *Physalis pubescens* ethyl acetate extracts (PHY-EA) on tumor cells.

Cell line	IC_50_ µg/mL
H2122	508.22 ± 152.72
HUH-7	120.11 ± 22.20
MDA-MB-231	113.88 ± 38.45
PC3	152.72 ± 15.66
SPC-A1	30.30 ± 3.03
HeLa	584.51 ± 16.49
HepG2	44.12 ± 17.07
B16	16.82 ± 5.62
SU-DHL-4	9.49 ± 0.12
CA-46	2.49 ± 0.05
Daudi	4.79 ± 0.60

Note: the above data were obtained from three independent experiments and are expressed as means ± standard errors (SE).

In contrast, the proliferation inhibitory effect of PHY-EA on the three types of normal cells (HSF, HaCat, and Raw264.7) was less than the effect against these lymphoma cells, with IC_50_ values higher 10 μg/mL (Figure 1C).

### Anti-tumor effect of physalin B, O, and L

We used SRB to screen for the anti-tumor activity of physalin B, physalin O, and physalin L. The screening results showed that physalin B had the best anti-tumor effect of the three compounds, with a proliferation inhibitory effect on a variety of tumor cells and the best effect on lymphoma cells CA-46 (IC_50_ = 4.78 ± 0.61 μg/mL). Based on the results of the HPLC assay, we hypothesized that the main active component of PHY-EA was physalin B (Figure 1D).

### PHY-EA induces apoptosis in lymphoma cells

**FIGURE 2:**
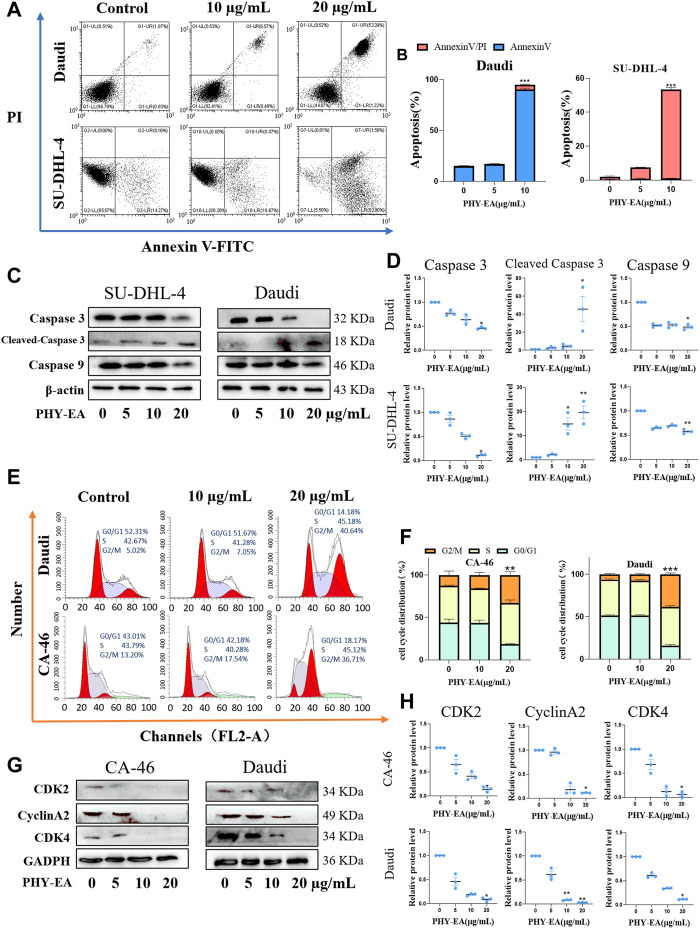
*Physalis pubescens* ethyl acetate extract (PHY-EA) induced apoptosis and blocked the cell cycle in lymphoma cells. **(A)** PHY-EA induced apoptosis in lymphoma cells. **(B)** Changes in the apoptosis rate of lymphoma cells induced by PHY-EA. **(C)** Western blot detection of caspase 9, caspase 3, and cleaved caspase 3 changes in lymphoma. **(D)** Normalized grayscale analysis of apoptosis-associated protein images. **(E)** Effect of PHY-EA on the cell cycle of lymphoma cells. **(F)** PHY-EA induced cell cycle changes in lymphoma cells. **(G)** Changes in cell cycle-related proteins. **(H)** Normalized grayscale analysis of cycle-related protein images. Data are expressed as means ± standard errors (SE), and each experimental group was compared with the control group using a Student’s *t*-test, with significance denoted by * for *p* < 0.05, ** for *p* < 0.01, and *** for *p* < 0.001.

Flow cytometry and Western blotting were performed to investigate the mechanism underlying the proliferation-inhibiting effect of PHY-EA on lymphoma cells. The apoptosis rate of Daudi and SU-DHL-4 cells induced by 20 μg/mL of PHY-EA increased by 79.79% and 51.49%, respectively, compared with that of the control group (Figures 2A,B). Western blot assay results showed that in the Daudi cells, the expression levels of caspase 3, cleaved caspase 3, and caspase 9 were 46.5% ± 2.6%, 4,568.1 ± 2,428.9%, and 48.3% ± 5.6%, respectively, compared with the control group. In addition, in the SU-DHL-4 cells, the expression levels of caspase 3, cleaved caspase 3, and caspase 9 were 10.6% ± 2.4%, 2,426.1 ± 1,489.3%, and 573% ± 45%, respectively, compared with the control group (Figures 2C,D). These results demonstrate that PHY-EA has a strong apoptosis-inducing effect on lymphoma cells.

Blocking effect of PHY-EA on the G2/M phase→G0/G1 phase of lymphoma cells.

The cyclic effect of PHY-EA on lymphoma cells was investigated using flow cytometry, and cycle-related proteins were examined using Western blot assays. Compared with the control group, 20 μg/mL of PHY-EA resulted in a 20.52% and 32.05% increase in the G2/M phase of Daudi and CA-46 cells, respectively (Figure 2F). The results of the Western blotting experiments in 20 μg/mL of PHY-EA showed that the expression levels of CDK2, cyclinA2, and CDK4 in the CA-46 cells were 14.0% ± 3.2%, 11.3% ± 0.1%, and 6% ± 3.5%, respectively, compared with the control group. Similarly, in the Daudi cells, the expression levels of CDK2, cyclinA2, and CDK4 were 8.7% ± 2.4%, 3.3% ± 0.3%, and 11.7% ± 0.3%, respectively (Figures 2G,H). This demonstrated that PHY-EA has a blocking effect on lymphoma cells in the G2/M→G0/G1 phase.

### PHY-EA inhibits lymphatic transplantation tumors in mice

**FIGURE 3:**
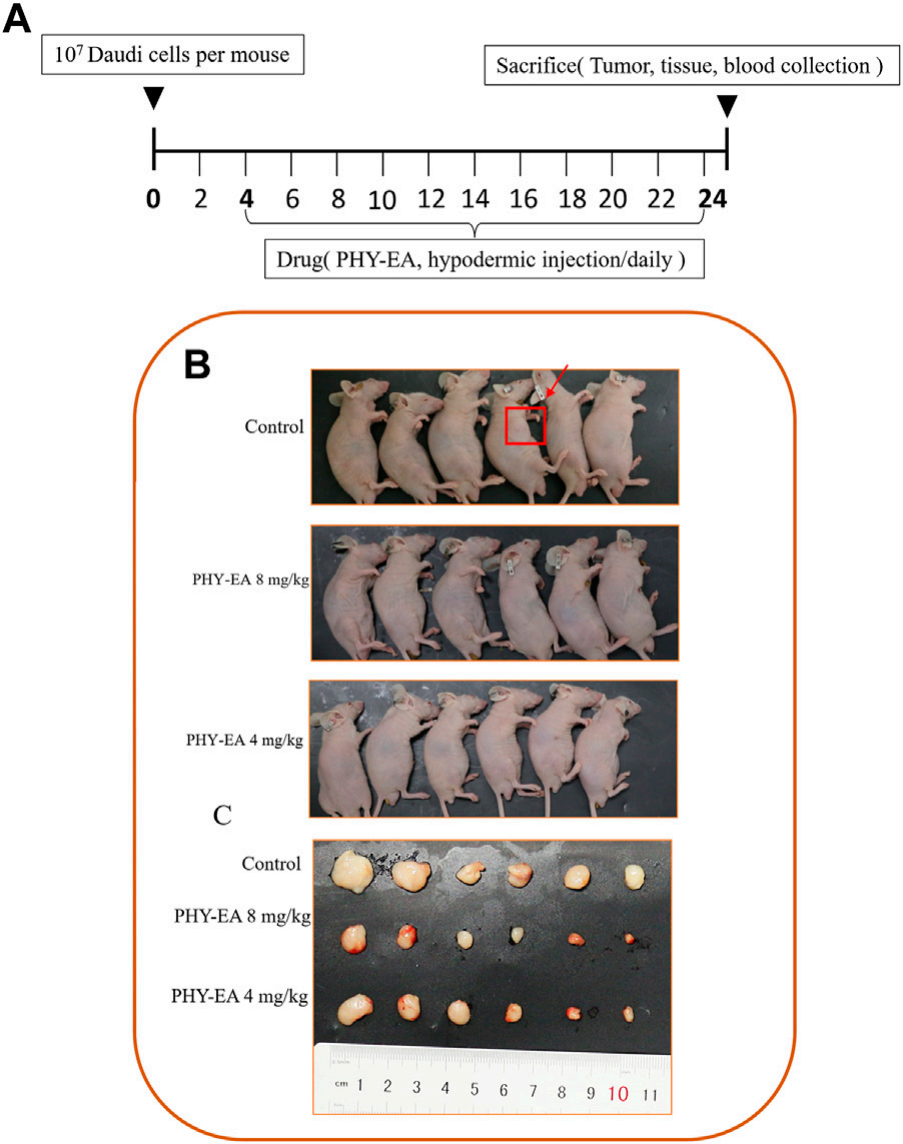
Mice with transplanted tumors. **(A)** Schematic diagram of the animal experimental method. Mice were divided into three groups: control group, *Physalis pubescens* ethyl acetate extract (PHY-EA) 8 mg/kg group, and PHY-EA 4 mg/kg group, with treatments administered for 21 days. **(B)** Pictures of transplanted tumor mice. **(C)** Pictures of transplanted tumors.

**FIGURE 4:**
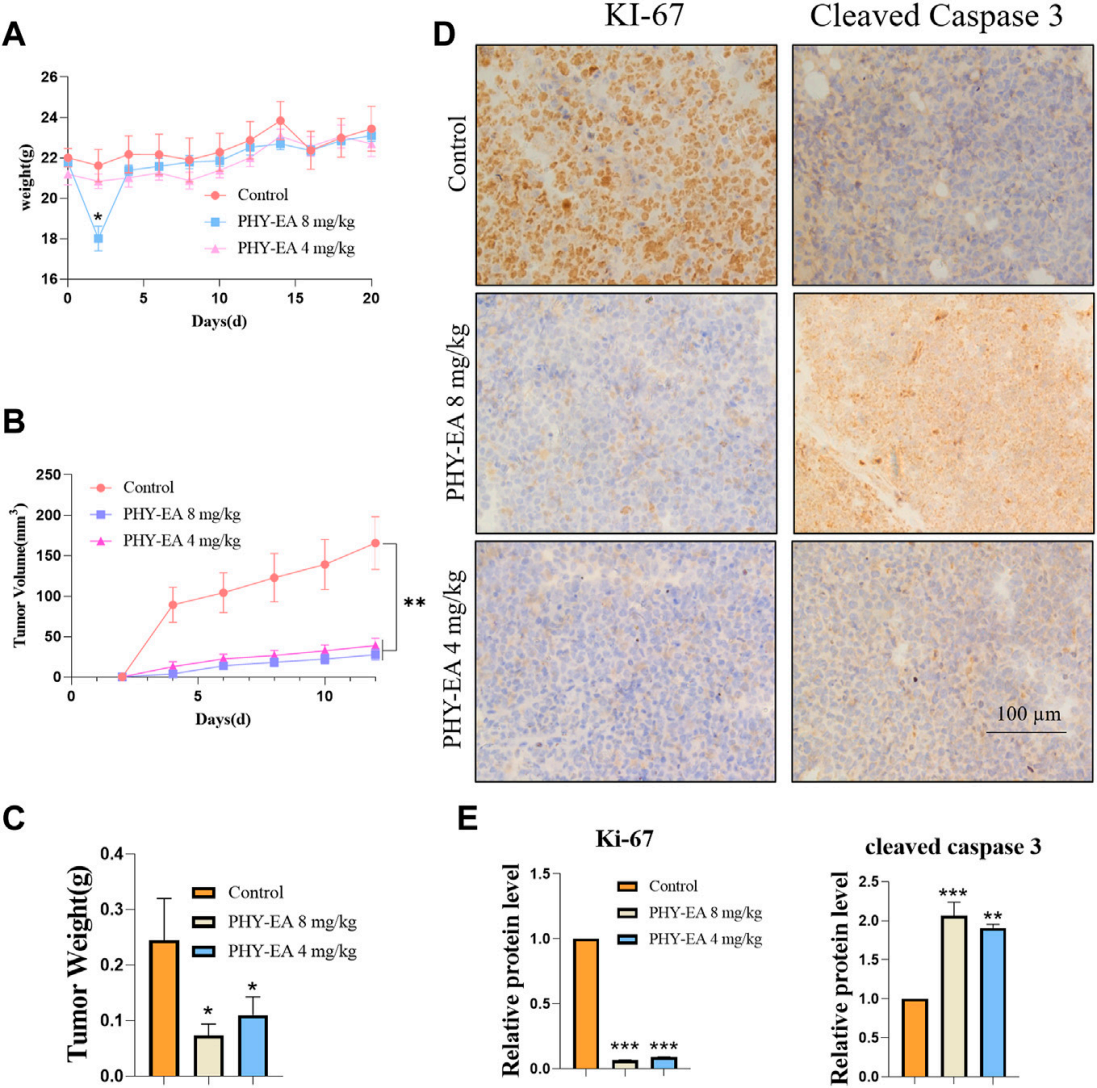
Tumor parameters and immunohistochemistry. **(A)** Body weight of mice. **(B)** Tumor volume. **(C)** Tumor weight. **(D)** Immunohistochemistry of Ki-67 and cleaved caspase 3 in tumor tissues. **(E)** Immunohistochemistry grayscale analysis. Data are expressed as means ± standard errors (SE), and each treatment group was compared with the control group using student's t tests, with significance denoted by * for *p* < 0.05, ** for *p* < 0.01, and *** for *p* < 0.001. PHY-EA, *Physalis pubescens* ethyl acetate extract.

A transplanted tumor assay was performed to study the anti-tumor activity of PHY-EA against lymphoma in vivo. Tumor cells were injected into the subcutaneous axilla of the mice and then treated with PHY-EA (Figures 3A,B). At the end of the experiment, the tumor weight was 0.24 ± 0.07 g in the control group, 0.07 ± 0.02 g in the PHY-EA 8 mg/kg group, and 0.11 ± 0.03 g in the PHY-EA 4 mg/kg group. The tumor volume in the control group was 165.4 ± 32.5 mm^3^, 27.6 ± 6.7 mm^3^ in the PHY-EA 8 mg/kg group, and 38.6 ± 6.7 mm^3^ in the PHY-EA 4 mg/kg group was. Compared with the control group, the tumor volume decreased by 83.2% and 72% in the PHY-EA 8 mg/kg group PHY-EA 4 mg/kg group, respectively (Figure 3C and Figure 4B). The body weights of mice in each group did not significantly differ during the experiment (Figure 4A).

Immunohistochemical assays were used to detect changes in the expression of proliferation-related proteins in the tumor tissues. The results showed that the relative expression of the Ki-67 protein was 0.066 ± 0.002 in the PHY-EA 8 mg/kg group and 0.088 ± 0.004 in the PHY-EA 4 mg/kg group, which were 93.4% and 91.2% lower than the control group, respectively. The relative expression of cleaved caspase 3 protein in the PHY-EA 8 mg/kg and PHY-EA 4 mg/kg groups was 2.16 ± 0.17 and 1.90 ± 0.04, respectively, an increase of 116% and 90% compared with the control group (Figures 4D,E). These results indicated that PHY-EA significantly inhibited the proliferation of mouse transplantation tumors by inducing apoptosis and cycle arrest in the tumor cells.

## Discussion

Physalin analogs are a group of highly oxidized 13,14-cleaved-ring-16,24-cycloergostane compounds found in *Physalis* species. More than 30 physalins have been isolated from the genus *Physalis* (Meira et al., 2022; Qu et al., 2022). We identified the components of PHY-EA as physalin B, physalin O, and physalin L—three physalin-like compounds, of which the Physalin B content was the highest at 1.8% ± 0.12%. The pharmacological activity of physalin B has been extensively reported, whereas the activities of physalin O and L have received little attention. We screened the *in vitro* anti-tumor activity of the three compounds and found that physalin B had higher anti-tumor activity, whereas physalin O and physalin L had poor proliferation inhibition. This result suggests that physalin B may be the main active component in PHY-EA and that structural differences between physalin B and physalin L may be a key factor affecting physalin activity. Meanwhile, the inhibition of cell proliferation by PHY-EA was lower in normal cells than some tumor cell lines and showed specific toxicity against lymphoma.

Using flow cytometry, PHY-EA was found to exert apoptosis-inducing and cycle-stopping effects on lymphoma. Apoptosis is a self-regulated form of cell death (Xu et al., 2019), and caspase family proteins play an important role in regulating apoptosis, including caspase 9, which can be signaled to self-activate and activate caspase 3, which activates CAD to degrade DNA and causes a series of apoptotic responses (Elmore, 2007). Our experiments demonstrated that PHY-EA can activate caspase 9 and thus caspase 3 to induce apoptosis in lymphoma cells.

The cell cycle is the process of cell proliferation and is divided into the G1, S, G2, and M phases depending on the DNA state (Schafer, 1998). Various cell cycle processes are regulated by different CDK and cyclin proteins (Coffman, 2004) (Engeland, 2018; Wang, 2022). Cyclin A activates CDK1 and CDK2 to promote transformation in the G2/M phase, and the CDK4/6-Cyclin D1 complex plays a key role in the G1 phase (Strzyz, 2016; Pai et al., 2021). The experimental results showed that PHY-EA regulates the cell cycle mainly by inhibiting Cyclin A and CDK2, thus inhibiting the G2/M phase transition process.

Despite these promising results, there is still a limitation in the current study, namely, the reasons for the specific activity of PHY-EA against lymphoma have not been fully explored. According to related studies (Ji et al., 2013; Xia et al., 2016), PHY-EA may contain a large number of physalin-like compounds, which are also responsible for the good anti-tumor activity of PHY-EA; however, we did not conduct a comprehensive isolation, identification, and activity study of other compounds in PHY-EA, which needs to be explored in our future research.

## Conclusion

PHY-EA, an extract of the leaves and twigs of *P. pubescens*, showed good anti-tumor activity against Burkitt’s lymphoma, demonstrating that in addition to the calyx and fruit, the twigs and leaves of *P. pubescens* also have medicinal value. Compared with the fruit, the leaves and branches of *P. pubescens* are less expensive, so their application will help reduce the costs associated with producing drugs from *P. pubescens*.

## Data Availability

The original contributions presented in the study are included in the article/supplementary materials, further inquiries can be directed to the corresponding authors.
